# Barriers of access to primary healthcare services by National Health Insurance Fund capitated members in Uasin Gishu county, Kenya

**DOI:** 10.1186/s12913-024-11282-8

**Published:** 2024-09-04

**Authors:** Barbara Nawire Were, Eunice Muthoni Mwangi, Lillian Wambui Muiruri

**Affiliations:** 1 Department of Health Systems Management, Kenya Methodist University-Nairobi, Nairobi, Kenya; 2 Department of Population Health - Medical College, Aga Khan University-Nairobi, Nairobi, Kenya

**Keywords:** Capitation, National Hospital Insurance Fund, Universal Health Coverage

## Abstract

**Purpose:**

The study identifies provision of primary healthcare services using the capitated health model as a prerequisite for promoting positive healthcare outcomes for a country’s population. However, capitated members have continued to face challenges in accessing primary healthcare services despite enrolment in the National Health Insurance Fund (NHIF). This study sought to determine if variables such as patient knowledge of the NHIF benefit package, NHIF Premium Payment processes, selecting NHIF capitated health facilities, and NHIF Communication to citizens’ influences access to primary healthcare services.

**Method:**

A cross-sectional analytical research design was adopted. Data was collected from patients who were using NHIF cards, who were drawn from health facilities. Data was collected using a structured questionnaire where some of the questions were rated using the Likert scale to enable the generation of descriptive statistics. Data was analysed using descriptive and inferential statistics. Logistic regression was conducted to determine the relationship between the independent and the dependent variables.

**Results:**

The study found that four independent variables (Patient knowledge of NHIF Benefit Package, NHIF Premium Payment processes, Selecting NHIF capitated Health Facility, and NHIF Communication to citizens) were significant predictors of access to capitated healthcare services with significance values of .001, .001, .001 and .001 respectively at 95% significance level.

**Conclusions:**

The study found that familiarity with the NHIF benefit package significantly influenced NHIF capitated members' access to primary healthcare services in Uasin Gishu County. While most members were aware of their healthcare entitlements, there's a need for increased awareness regarding access to surgical services and dependents' inclusion. Facility selection also played a crucial role, influenced by factors like freedom of choice, NHIF facility selection rules, facility appearance, and proximity to members' homes. NHIF communication positively impacted access, with effective communication channels aiding service accessibility. Premium payment processes also significantly linked with service access, influenced by factors such as payment procedures, premium awareness, payment schedules, registration waiting periods, and penalties for defaults. Overall, patient knowledge, NHIF communication, premium payment processes, and facility selection all contributed positively to NHIF capitated members' access to primary healthcare services in Uasin Gishu County.

**Supplementary Information:**

The online version contains supplementary material available at 10.1186/s12913-024-11282-8.

## Introduction

Health financing plays a critical role in the functioning of healthcare systems, encompassing the acquisition, pooling, and allocation of funds to address people’s healthcare needs [1]. An effective health financing system should be capable of consistently generating sufficient funds to facilitate access to high-quality health services without imposing financial burdens [2, 3]. The core functions of health financing comprise revenue collection, risk pooling, and the purchasing of health services [4, 5]. Purchasing can take either a passive or strategic approach. Strategic purchasing aims to optimize health system performance, while passive purchasing operates within predetermined budgetary constraints. Strategic purchasing, integral to achieving Universal Health Coverage (UHC), prioritizes both adequate resource mobilization and efficient resource utilization [6–8]. Provider payment mechanisms within healthcare systems primarily include fee-for-service, salary, and capitation. Strategic employment of capitation holds promise for advancing UHC objectives. Capitation involves providers receiving fixed payments per enrolled individual over a defined period, fostering a shift towards preventive care [9]. Simplified billing procedures under capitation streamline administrative processes and incentivize preventive healthcare, thus reducing reliance on costly interventions [10–12].

Globally, the capitation model predominates in tax-funded health systems like those of Italy and the UK, where general practitioners deliver primary care [13, 14]. As countries commit to UHC, many are considering integrating capitation with other payment models to enhance healthcare efficiency and performance [13, 15]. However, challenges such as inadequate incentive structures and concerns about limiting consumer choice hinder widespread adoption [16, 17].

Kenya's National Hospital Insurance Fund (NHIF) predominantly employs capitation to finance primary healthcare services, contracting various organizations to provide outpatient care. Despite the theoretical benefits of capitation in promoting preventive care and reducing hospitalization rates, challenges persist, including inadequate funding and delays in payment [18–20]. Kenya's efforts to achieve UHC through NHIF expansion face obstacles such as reliance on out-of-pocket payments and inconsistencies in service provision [21–23].

To address these challenges, this study was conducted in Uasin Gishu County, Kenya, to explore the barriers of access to primary healthcare services by national health insurance fund capitated members in Uasin Gishu county, Kenya. The findings aim to inform decision-makers and stakeholders on measures to enhance access to primary healthcare services within the national scheme.

## Methods

This cross-sectional analytical research was conducted in Uasin Gishu County, Kenya, chosen due to its large population exceeding 1.3 million in 2019 and a notable inter-censual growth rate of 3.6%, surpassing the national rate of 2.9% [24]. The county's poverty rate, at 44.6% as of 2006, highlights socio-economic disparities affecting the purchasing power of primary healthcare services. Among its 125 public health facilities, there's one national referral hospital, two district hospitals, 11 sub-district hospitals, 88 dispensaries, and 23 health centres. Utilizing a multistage sampling technique, 90 facilities were selected, targeting over 10,000 capitated members [25]. Based on Krejcie and Morgan's formula [14], a sample of 384 respondents was determined, proportionately allocated to the facilities according to NHIF registration. Within each facility, simple random sampling was employed.

Data collection utilized a structured questionnaire, employing a three-point Likert scale, administered in English, covering socio-demographic characteristics, independent variables (NHIF benefit package, premium payment processes, communication to citizens, and healthcare provider selection), and the dependent variable (access to NHIF primary healthcare services) [26]. Logistic regression analysed the relationship between independent and dependent variables, with Likert scale responses converted into binary variables, where agreement was coded as 1 and disagreement or not sure as 0. This binary coding facilitated the analysis, reflecting patients' access or lack thereof to primary healthcare services.

## Results

Two hundred eighty-two out of 384 participants responded, contributing to a 73% response rate, ideal for analysing socio-demographic characteristics such as gender, age, number of children, marital status, education, employment, household income, and NHIF contributions as presented in Table 1.

**Table 1:** Socio-Demographic Characteristics of the sample (*n* = 282)

Description	Frequency (n)	Percentage (%)
**Gender**		
Male	143	51
Female	139	49
**Age**		
18–24	31	11
25–34	124	44
35–44	43	15
45–54	19	7
55–64	6	2
Above 65	1	
**Marital Status**		
Single	111	39
Married	130	46
Cohabiting	19	7
Separated	12	4
Divorced	3	1
Widowed	5	2
**Number of Children**		
None	43	15
1–2	163	58
3–4	18	6
**Level of Education**		
None	4	1
Primary	11	4
Secondary	55	20
College	129	46
University	80	28
**Employment**		
Employed	126	45
Self Employed	123	43
Student	31	11
**Monthly Household Income (KShs.)**		
Less than 10,000	71	25
10,001–20,000	62	22
20,001–30,000	68	24
30,001–40,000	39	14
40,001–50,000	15	5
Above 50,001	20	7
**NHIF Monthly premium (KShs.)**		
None	3	1
100–500	125	44
501–1000	66	23
1001–1500	13	5
1501–2000	19	6
Above 2000	5	1

The primary age group of respondents was 25 to 34 years, typically productive and focused on personal and organizational growth. They exhibited a high likelihood of seeking healthcare services, with many having young families, leading to increased capitation subscription, contribution, and utilization rates. The majority had partners, had 1–2 children, had acquired tertiary education, had employment, and had a household income adequate for NHIF premiums.

### Access to NHIF primary care health services

The study dependent variable was patient’s access to NHIF Primary Care Health Services. Descriptive results are presented in Table 2 [27].

**Table 2:** Access to NHIF Primary Care Health Services

Statement	Disagree	Agree
	**n (%)**	**n (%)**
i. Service providers are always willing to the help patient	79(28)	203(72)
ii. Service providers give patients personal attention	92(32)	190(67)
iii. I feel safe while interacting with the hospital employees	72(26)	210(74)
iv. Attitude of the service providers is good	72(25)	210(75)
v. I get all prescribed drugs and services at the facility	135(48)	147(52)
vi.The staff are trained and qualified	79(28)	203(72)
vii. I have access to all NHIF outpatient services	97(34)	185(66)
viii. The waiting time is often not too long	111(39)	171(61)
ix. NHIF prescribed services are always available	109(39)	173(61)
x. Sometimes I am asked to co-pay for registration, consultation, medications, or laboratory services	56(20)	226(80)

Over 70% agreement was observed regarding positive staff attitude, feeling safe while with staffs, staff competence and willingness to assist patients, NHIF service availability, and manageable waiting times. However, 226(80%) agreed to paying out-of-pocket for registration, consultation, medications, or laboratory services despite having prepaid for the services.

### Logistic regression

The independent variable in this study were (NHIF Benefit Package, premium payment processes, communication to citizens, and healthcare provider selection) and the dependent variable was access to NHIF primary healthcare services. Logistic regression was undertaken of the variables to determine the barriers of access to primary healthcare services by national health insurance fund capitated members in Uasin Gishu county, Kenya.

The model used in this study was as follows:

$$f\left(z\right)=\frac{1}{1+{{e}^{-}}^{Z}}$$

where Z is a linear combination of the covariates expressed as:

The model employed in this study was formulated as follows:

$$Z = \beta 0 + \beta 1X1 + \beta 2X2 + \beta 3X3 + \beta 4X4$$

where Z represents a linear combination of the covariates, with X1, X2, X3, and X4, being the independent variables (NHIF Benefit Package, premium payment processes, communication to citizens, and healthcare provider selection). The intercept is represented by β0, whereas β1, β2, β3 and β4 denote the estimates of the increase in log odds of the dependent variable (access to NHIF primary care health services) for each unit increase in the respective independent variables. An odds ratio of 1 indicates that the independent variable has no effect on the dependent variable. An odds ratio greater than one suggests a greater risk association, while ratio less than one indicates a reduced risk or the ability of the independent variable to mitigate the risk of access to NHIF primary care health services [28].

A logistic regression analysis was conducted to examine the impact of patients’ knowledge of the NHIF benefit package, premium payment process, facility selection, and communication from NHIF on their access to services. The results are presented in Table 3 [29]

**Table 3:** Logistic regression results

Variable	B	S.E	Odds Ratio	*P-value*
**NHIF benefit package**				
Patients don’t know benefit package (ref)			1.000	
Patients know benefit package	2.227	0.688	9.274	0.001
**NHIF premium payment**				
Patients don’t know premium payment process (ref)			1.000	
Patients know premium payment process	1.675	0.607	5.339	0.006
**Selecting health facilities**				
Patients don’t know how to select facility (ref)			1.000	
Patients know how to select facility	1.809	.414	6.101	0.001
**NHIF communication to citizens**				
Patient don’t receive communication (ref)			1.000	
Patients receive communication	1.360	.413	3.896	0.001

The results reveal that patients who knew the NHIF benefit package were 9.274 times more likely to receive the healthcare services compared to those unaware of the benefit package. In addition, patients who knew about the NHIF premium payment process were 5.339 times more likely to visit the NHIF recommended health facilities compared to those unaware of the process. The NHIF members who knew how to select a health facility were 6.101 times willing to access NHIF services compared to their counterparts who had no knowledge of health care facility selection. Members who receive communication were also more likely to access primary healthcare services.

## Discussion

The predominant age group among respondents was between 25 and 34 years, representing individuals at their peak productivity and deeply invested in personal and professional growth. This cohort exhibited the highest likelihood of seeking healthcare services, leading to increased rates of capitation subscription, contribution, and utilization [30]. Married couples showed a higher rate of insurance coverage, attributed to their responsibilities towards dependents and a comparatively higher household income, facilitating premium payments [31]. Moreover, a majority of respondents had attained at least minimum academic and professional qualifications, enhancing their understanding of NHIF procedures and terms. They also boasted household incomes adequate for monthly NHIF contributions [32].

However, patient awareness of NHIF primary health service benefits remained limited, with approximately 25% expressing disagreement or uncertainty regarding their entitlements [23, 33]. Understanding of NHIF benefits significantly influenced access to capitated health services positively [34]. While NHIF continuously reviews its benefit package, many members only become aware of the outpatient services when they necessitate them, possibly due to the on-demand nature of outpatient care utilization [35, 36].

Effective communication from NHIF positively impacted access to primary care health services under capitation. Despite positive feedback regarding NHIF's provision of necessary information, a significant portion of respondents expressed dissatisfaction with NHIF's responsiveness to public complaints and its clarity regarding service packages. NHIF primarily communicates through its website and media advertisements, but the limited reach of these channels potentially hinders members' awareness of their entitlements, affecting healthcare access and potentially leading to under or over-provision of services [18, 23, 37–39]. Equity and efficiency in healthcare provision can be achieved by empowering the service providers and the members with the information by adopting effective communication channels [40, 41]. The findings agree with other studies which show that some of the communication media used by the NHIF included television, radio, newspaper, social media, mobile phones, billboards, and sensitization campaigns [33, 42]. They agree with the findings of another study where reportedly 57% of the respondents are provided information by NHIF while 43% are not receiving any communication from NHIF regarding the health services covered [23, 33, 43]. In addition, the results also highlight that no legislation provides for feedback or complaints mechanism from members or beneficiaries [18, 23, 44].

Approximately 20% of respondents were unaware of the premium payment process, indicating a lack of clarity regarding NHIF contributions [45–48]. The perception of NHIF-accredited health facilities significantly influenced service utilization, with patients associating the facilities' image with service quality [35, 36, 49]. However, the accreditation status varied among clinics and higher-level facilities, potentially impacting service uptake [50, 51]. Additionally, individuals diagnosed with chronic illnesses exhibited a higher likelihood of selecting healthcare providers and utilizing services, with private providers restricted to specific service categories compared to government hospitals [52, 53].

### Limitations

Using structured questions to collect self-reported data. Participants may provide inaccurate or biased responses due to social desirability bias or recall bias. Additionally, respondents' comprehension of the questions or their willingness to disclose certain information could vary, leading to inconsistencies in the data collected. This could affect the validity and reliability of the study's findings.

The findings of the research may have limited generalizability beyond the specific context of Uasin Gishu County. Factors influencing access to primary healthcare services can vary significantly depending on geographical location, cultural norms, healthcare infrastructure, and other contextual factors. Therefore, the determinants identified in this study may not be applicable to capitated members in other regions of Kenya or in different counties; limiting the broader applicability of the research findings and the study also targeted NHIF accredited public health facilities in Uasin Gishu County, Kenya. Privately owned hospitals were excluded. Hence the study findings can be generalized to public facilities.

## Conclusions

Out of pocket payment despite prepayment remains a key barrier of access to primary healthcare services. NHIF capitated members are generally aware of their healthcare entitlement. However, efforts are needed to enhance more awareness regarding entitlement to surgical services, inclusion of dependents, access to information about the benefit package, feedback and complaint mechanisms, premium payment process, awareness of the premium to be paid, payment schedule, the waiting period before registration and accessing services, and penalties in the event of default. This information could be used to advocate for the implementation of effective communication systems that allow for real-time dissemination of information and feedback, as well as to conduct regular in-service training and recruiting a well-educated workforce that is familiar with NHIF procedures and terms in order to promote the NHIF in the face of capitated members' social-demographic profiles, and to increase knowledge of the health coverage plan for the informal sector and flexible payment platforms. Utilizing vernacular to reach more communities, particularly in rural areas could broaden outreach.

## Supplementary Information

Supplementary Material 1.

Supplementary Material 2.

## Data Availability

The datasets used and/or analysed during the study are available from the corresponding author on reasonable request.
