# Supplementary material for: Barriers of access to primary healthcare services by National Health Insurance Fund capitated members in Uasin Gishu county, Kenya
Source: BMC Health Serv Res. 2024 Sep 4;24:1025. doi: 10.1186/s12913-024-11282-8 (PMC11375832; doi:10.1186/s12913-024-11282-8)
Supplement: Supplementary file 1 — Supplementary Material 1. [file 12913_2024_11282_MOESM1_ESM.docx]

### Patient perception of NHIF Benefit package, NHIF Communication to citizens, Selecting Healthcare Provider and Premium payment process

| **NHIF Primary Health Service Benefits** | **Disagree** | **Not Sure** | **Agree** |
| --- | --- | --- | --- |
|  | **n(%)** | **n(%)** | **n(%)** |
| 1. General Consultation | 23(09) | 07(02) | 252(89) |
| 1. Basic Lab Investigations | 23(09) | 29(10) | 230(82) |
| 1. Prescription & administration of drugs | 30(10) | 24(09) | 228(81) |
| 1. Minor surgical procedures | 44(15) | 28(10) | 210(74) |
| 1. Inclusion of dependents | 34(12) | 38(13) | 210(74) |
| **NHIF Communication to Citizens** |  |  |  |
| 1. NHIF communicates to me regularly through SMS, Newspaper, Radio, TV | 54(19) | 18(06) | 210(75) |
| 1. NHIF uses a language I understand | 22(07) | 15(05) | 245(87) |
| 1. NHIF guides me in the process of registration | 44(16) | 43(15) | 195(69) |
| 1. NHIF provides me with all the information I require and need to know | 47(17) | 49(17) | 186(66) |
| 1. NHIF states the range of services offered in each benefit package | 53(19) | 38(13) | 191(68) |
| 1. NHIF always responds to public complaints and feedback | 93(33) | 45(16) | 144(51) |
| **Selecting Primary Healthcare Provider** |  |  |  |
| 1. NHIF communicates the rules for selecting a healthcare facility | 36(12) | 38(13) | 208(74) |
| 1. NHIF provides an adequate number of health facilities for patients to choose from | 54(19) | 39(14) | 189(67) |
| 1. I can choose more than one facility under NHIF contracted facilities | 74(26) | 41(15) | 167(59) |
| 1. I choose the facility at my free will | 35(12) | 33(12) | 214(76) |
| 1. The facility is close to my home | 55(19) | 23(08) | 204(72) |
| 1. Physical facilities are visually appealing | 41(15) | 29(10) | 212(75) |
| **Premium Payment Process** |  |  |  |
| 1. Am aware of the amount of monthly premium paid | 15(05) | 19(07) | 248(88) |
| 1. Am aware of the process of paying NHIF contributions | 18(07) | 26(09) | 238(84) |
| 1. There are penalties in case of defaulting NHIF premiums for 3 months | 41(15) | 45(16) | 196(70) |
| 1. Premium payment schedule is on 10^th^ of every month. | 27(10) | 26(09) | 229(81) |
| 1. Waiting period to access primary care services after registration | 41(15) | 40(14) | 201(71) |

The study evaluated respondents' perception of the NHIF Benefit package, focusing on outpatient benefits like general consultations, basic lab investigations, prescriptions, minor surgical procedures, and inclusion of dependents. Majority of the respondents 252(89%) agreed that general consultation was one of the benefits, followed by basic lab investigations 230(82%), prescriptions and administration of drugs 228(81%), treatment for local diseases, 227(80%) and minor surgical procedures and inclusion of dependents 210(74%).

Most respondents 245(87%) acknowledged NHIF's use of understandable language in communication, with regular updates 210(75%) through SMS, newspapers, radio, and TV. Additionally, 195(69%) agreed that NHIF assists in registration, 191(68%) specifies service packages, 186(66%) provides necessary information, and 144(51%) addresses public feedback.

Most respondents 218(76%) affirmed their ability to freely select registered primary healthcare providers, finding facilities visually appealing 212(75%), understanding selection rules 208(74%), and appreciating proximity to their homes 204(72%). However, a minority 74(26%) disagreed with selecting multiple facilities, and some 54(19%) felt NHIF didn't offer sufficient options for selecting healthcare facilities.

.

The NHIF Premium Payment processes, including monthly premiums, payment procedures, penalties for defaulting, payment schedule, waiting period after registration, and access to health services, were evaluated. Results showed strong agreement across variables, with the highest scores for monthly premiums 248(88%), payment procedures 238(84%), and payment schedule 229(81%). However, knowledge of waiting periods 201(71%) and defaulting penalties 196(70%) scored lower.
