# Supplementary material for: Barriers of access to primary healthcare services by National Health Insurance Fund capitated members in Uasin Gishu county, Kenya
Source: BMC Health Serv Res. 2024 Sep 4;24:1025. doi: 10.1186/s12913-024-11282-8 (PMC11375832; doi:10.1186/s12913-024-11282-8)
Supplement: Supplementary file 2 — Supplementary Material 2. [file 12913_2024_11282_MOESM2_ESM.docx]

## Appendix 1: Informed Consent Letter

Kenya Methodist University

P. 0 Box 267-60200

MERU, Kenya

**SUBJECT: INFORMED CONSENT**

**Dear Respondent**,

My name is Barbara Were, I am a master’s student from Kenya Methodist University. I am conducting research titled: **Determinants of Primary Healthcare Services Access by National Health Insurance Fund (NHIF) Capitated members in Uasin Gishu County, Kenya.** The findings will be used to strengthen the health systems in Kenya and other low and middle-income countries in Africa as a result, counties, communities, and individuals will also benefit from it.

The survey will take about 40 minutes to complete, and I would be glad if you would kindly give your consent to participate in the study and answer all the questions correctly. The answers you provide will be kept confidential and anonymous and would only be used for the research.

**Procedure to be followed**

Participation in this study will require you to answer the questions on the questionnaire which will be provided. You have the right to refuse participation in this study. You will not be penalized nor victimized for not joining the study and your decision will not be used against you nor affect you as you seek healthcare services. Please remember that participation in the study is voluntary. You may ask questions related to the study at any time. You may refuse to respond to any questions, and you may stop an interview at any time. You may also stop being in the study at any time without any consequences to the services you are rendering.

**Rewards**

There is no reward for anyone who chooses to participate in the study.

**Confidentiality**

The questionnaire will be given in a private setting. Your name will not be recorded on the questionnaire and the questionnaires will be kept in a safe place at the University.

**Contact Information**

If you have any questions, you may contact the following supervisors:

1. Dr. Eunice Muthoni Mwangi

Department of Population Health

Aga Khan University Nairobi - East Africa.

Mobile No. 0722 986 349

2. Ms. Lilian Muiruri

Department of Health Systems Management

Kenya Methodist University, Nairobi campus.

Mobile No. 0724 956 049

**Participant’s Statement**

The above statement regarding my participation in the study is clear to me. I have been given a chance to ask questions and my questions will be answered to my satisfaction. My participation in this study is entirely voluntary. I understand that my records will be kept private and that I can leave the study at any time. I understand that I will not be victimized at my place of where I seek healthcare services whether I decide to leave the study or not and my decision will not affect the way I am treated at the healthcare facility.

Name of Participant…………………………………………………Date………………………….

Signature………………………………………….

**Investigator’s Statement**

I, the undersigned, have explained to the volunteer in a language s/he understands the procedures to be followed in the study and the risks and the benefits involved.

Name of Interviewer………………………………………………….Date……………………

Interviewer Signature…………………………………………

## Appendix 2: Questionnaire for Citizens registered under the NHIF National Scheme

Date…...…Month……...………...Year……....

**Section A; Socio-demographic characteristics**

1. **Sex/Gender**

- Male
- Female

1. **What is your age? (years)**
2. **What is your marital status?**

- Single
- Married
- Cohabiting
- Separated
- Divorced
- Widowed

1. **How many children do you have?**
2. **What is the highest level of education attained?**

- None
- Primary
- Secondary
- Certificate
- Diploma
- Graduate
- Postgraduate
- Any other (specify)

1. **What is your occupation?**

- Employed
- Self-employed
- Student
- Any other (specify)

1. **What is your monthly household income in Kshs?**

- Less than 10,000
- 10,001 – 20,000
- 20,001 – 30,000
- 30,001 – 40,000
- 40,001 – 50,000
- 50,001 and above

1. **How much do you contribute per month? (Kshs)**

**Section B; Patient knowledge of the NHIF National scheme**

**Strongly Agree - SA, Agree - A, Not Sure - NS, Disagree – D, Strongly Disagree- SD**

| **I am always entitled to the following Benefit Package** | **SA** | **A** | **NS** | **D** | **SD** |
| --- | --- | --- | --- | --- | --- |
| 1. General consultation |  |  |  |  |  |
| 1. Basic lab investigations |  |  |  |  |  |
| 1. Prescription and administration of drugs |  |  |  |  |  |
| 1. Treatment for local diseases |  |  |  |  |  |
| 1. Minor surgical procedures |  |  |  |  |  |
| 1. Inclusion of dependents |  |  |  |  |  |
| **I am aware of the following on.**  **Payment of premiums** |  |  |  |  |  |
| 1. Amount of monthly contributions needed |  |  |  |  |  |
| 1. Process of how to pay the contributions |  |  |  |  |  |
| 1. Penalties in case of defaulting for 3 months |  |  |  |  |  |
| 1. Payment schedule (monthly, quarterly, twice a year, annually) |  |  |  |  |  |
| 1. Waiting period before registration and accessing services |  |  |  |  |  |
| **Selecting a Healthcare facility** |  |  |  |  |  |
| 1. NHIF communicates the rules for selecting a healthcare facility |  |  |  |  |  |
| 1. NHIF provides an adequate number of health facilities for patients to choose from |  |  |  |  |  |
| 1. I can choose more than one facility under NHIF |  |  |  |  |  |
| 1. I choose the facility at my free will |  |  |  |  |  |

**SECTION C; NHIF Information Sharing Mechanisms**

**Strongly Agree - SA, Agree - A, Not Sure - NS, Disagree – D, Strongly Disagree- SD**

| **To what extent do you agree with the following statements** | **SA** | **A** | **NS** | **D** | **SD** |
| --- | --- | --- | --- | --- | --- |
| 1. NHIF communicates to me regularly through SMS, Newspaper, Radio, TV |  |  |  |  |  |
| 1. NHIF uses a language I understand |  |  |  |  |  |
| 1. NHIF guides me in the process of registration |  |  |  |  |  |
| 1. NHIF provides me with all the information I require and need to know |  |  |  |  |  |
| 1. NHIF states the range of services offered in each benefit package |  |  |  |  |  |
| 1. NHIF always responds to public complaints and feedback |  |  |  |  |  |

**Section D; Patient Health - Seeking behaviour and access to capitated health services**

**Strongly Agree - SA, Agree - A, Not Sure - NS, Disagree – D, Strongly Disagree - SD**

| **Perception of Health Service Quality** | **SA** | **A** | **NS** | **D** | **SD** |
| --- | --- | --- | --- | --- | --- |
| 1. Physical facilities are visually appealing |  |  |  |  |  |
| 1. Service providers are always willing to the help patient |  |  |  |  |  |
| 1. Service providers give patients personal attention |  |  |  |  |  |
| 1. I feel safe while interacting with the hospital employees |  |  |  |  |  |
| **Preferences of Healthcare facilities** |  |  |  |  |  |
| 1. Attitude of the service providers is good |  |  |  |  |  |
| 1. I get all prescribed drugs and services at the facility |  |  |  |  |  |
| 1. The staff are trained and qualified |  |  |  |  |  |
| 1. The facility is close to my home |  |  |  |  |  |
| 1. I have access to all NHIF outpatient services |  |  |  |  |  |
| 1. The waiting time is often not too long |  |  |  |  |  |
| 1. NHIF prescribed services are always available |  |  |  |  |  |
| 1. Sometimes I am asked to co-pay for registration, consultation, medications, or laboratory services |  |  |  |  |  |
| **Awareness about having a disease** |  |  |  |  |  |
| 1. I am fully aware of my patient’s rights with regard to NHIF membership |  |  |  |  |  |
| 1. I am always aware when I need to seek treatment at the hospital |  |  |  |  |  |
| 1. I often go to seek treatment only when my condition gets worse |  |  |  |  |  |
| 1. I often self-medicate |  |  |  |  |  |
| 1. I often seek advice from friends, family, and neighbours’ before seeking professional advice |  |  |  |  |  |
